# IO-VNBD: Inertial and Odometry benchmark dataset for ground vehicle positioning

**DOI:** 10.1016/j.dib.2021.106885

**Published:** 2021-02-15

**Authors:** Uche Onyekpe, Vasile Palade, Stratis Kanarachos, Alicja Szkolnik

**Affiliations:** a Institute for Future Transport and Cities, Coventry University, Coventry, United Kingdom; b Faculty of Engineering and Computing, Coventry University, Coventry, United Kingdom; c Research Center for Data Science, Coventry University, Coventry, United Kingdom; d Coventry University, Gulson Road, Coventry, United Kingdom

**Keywords:** INS, Wheel odometry, Autonomous driving, GPS loss, Vehicular navigation, Vehicle positioning, Deep learning

## Abstract

Low-cost Inertial Navigation Sensors (INS) can be exploited for a reliable solution for tracking autonomous vehicles in the absence of GPS signals. However, position errors grow exponentially over time due to noises in the sensor measurements. The lack of a public and robust benchmark dataset has however hindered the advancement in the research, comparison and adoption of recent machine learning techniques such as deep learning techniques to learn the error in the INS for a more accurate positioning of the vehicle. In order to facilitate the benchmarking, fast development and evaluation of positioning algorithms, we therefore present the first of its kind large-scale and information-rich inertial and odometry focused public dataset called IO-VNBD (**I**nertial **O**dometry **V**ehicle **N**avigation **B**enchmark **D**ataset). The vehicle tracking dataset was recorded using a research vehicle equipped with ego-motion sensors on public roads in the United Kingdom, Nigeria, and France. The sensors include a GPS receiver, inertial navigation sensors, wheel-speed sensors amongst other sensors found in the car, as well as the inertial navigation sensors and GPS receiver in an Android smart phone sampling at 10 Hz. A diverse number of driving scenarios were captured such as traffic congestion, round-abouts, hard-braking, etc. on different road types (e.g. country roads, motorways, etc.) and with varying driving patterns. The dataset consists of a total driving time of about 40 h over 1,300 km for the vehicle extracted data and about 58 h over 4,400 km for the smartphone recorded data. We hope that this dataset will prove valuable in furthering research on the correlation between vehicle dynamics and dependable positioning estimation based on vehicle ego-motion sensors, as well as other related studies.

## Specifications Table

**Table:** 

Subject	Automotive Engineering, Signal Processing, Artificial Intelligence
Specific subject area	Positioning and Tracking of Autonomous Vehicles
Type of data	Excel csv
How data were acquired	Equipment • Racelogic VBOX Video HD2 CAN – Bus Data Logger (10 Hz) [15] • Racelogic VBOX Video HD2 GPS Antenna (10 Hz) [1] • Huawei P20 pro, Motorola moto G7 power and Blackberry Priv using AndroSensor Application (10 Hz) [2].
Data format	Raw
Parameters for data collection	The data was collected under a diverse number of environmental scenarios and vehicle motion states. The number of scenarios considered include bumps, hard braking, wet roads etc. See Table 4 for the full list of scenarios considered.
Description of data collection	The data was collected using four vehicles employing the sensors on a smartphone, GPS receiver and the sensors present in the sensor cluster of the vehicle. The smartphone data is sampled at 10 Hz with a GPS update rate of 1 Hz providing a total data size of about 2.2 million x 24, while the ECU recorded data is also sampled at 10 Hz with a total data shape of about 1.4 million x 29.
Data source location	Country: England, France, Nigeria Latitude and longitude (and GPS coordinates) for collected samples/data: GPS co-ordinates are provided in the dataset.
Data accessibility	Repository name: Github.com Data identification number: 2005.01701 Direct URL to data: https://github.com/onyekpeu/IO-VNBD
Related research article	U. Onyekpe, V. Palade, and S. Kanarachos, “*Learning to Localise Automated Vehicles in Challenging Environments using Inertial Navigation Systems (INS)” Applied Sciences 2021, 11*(3), 1270, https://doi.org/10.3390/app11031270

## Value of the Data

• The dataset is large-scale and diverse, and it focuses on inertial vehicle navigation under complex environmental scenarios and vehicle motion states such as varying longitudinal accelerations, hard-brakes, yaw rates, velocities, mud roads, motorways, etc. (see Table 4). The dataset consists of measurements from a rich combination of ego-motion sensors such as accelerometers, gyroscope, magnetometers, wheel encoders, force sensors, etc.
• The data is useful to research institutions and industries in the benchmarking, fast development, evaluation and testing of vehicle positioning and tracking algorithms and techniques.
• The data is useful for the robust training of supervised learning algorithms in learning the correlation between the dynamics of vehicles and their displacement, with applications in the tracking or positioning of vehicles and robots in GPS deprived environments using noisy low-cost sensors.

## 1 Data Description

The total dataset consists of about 100 h of recorded driving data on public roads by 8 different drivers with different driving styles as defined on Table 1, where defensive driving refers to situations where the vehicle is turned at less than 0.3 g, swerved at less than 3.3 km/hr or decelerated at less than 0.3 g, whilst aggressive driving refers to respective situations above these thresholds [3]. The data is divided into sets based on cities and towns driven via, road conditions, weather conditions, driving length and time, driving style and driving features (see Tables A1-1 to A6). The dataset also contains more than 20 min of data recorded from the stationary vehicle to aid in the estimation of the sensors’ bias. To add to the diversity of the data consisting of a number of complex driving scenarios as shown on Table 4, the data was recorded with different tyre pressures. Datasets with each unique tyre pressures are indicated on Tables A1-1 to A5-2 using Table 2 as a guide. Tables A1-1 to A6 reveal more detailed information on each set of the data. The data logged from the vehicle's CAN bus are denoted with the prefix “*V-*” and the smartphone data denoted with the prefix “*S-*”. The “*S-*” datasets are acquired from the sensors in a smartphone attached to the vehicle mimicking its motion.^1^ While all the “*V-*” datasets were collected only in England, the “*S-*” *datasets* were collected in England, France and Nigeria.

Over the course of the data collection, communication difficulties between the GPS receiver and satellites were encountered. Information on data indexes recorded during these periods are provided in a file titled “*GPS outages*”. Where possible, the “*S-*” and “*V-*” datasets which were collected simultaneously,^2^ are manually synchronised and stored in the folder named “*Synchronised V and S datasets”.*

Importantly, despite the effort lent towards an accurate alignment of the smartphone's sensor axis with that of the vehicle, the precision of the measurements were interfered by vehicular vibrations averagely estimated to be about 0.15 g of acceleration and 0.08 rad/s of yaw rate particularly at peculiar scenarios such as hard brakes or over bumps. Information on the amount of gravitational acceleration measured by each of the three axis are provided in the “S-” datasets to help in the correction of the measured acceleration. The data is stored in csv format at https://github.com/onyekpeu/IO-VNBD along with useful Python development tools.

**Table 1:** Driving pattern of each driver.

Driver	Driving Style
**A**	Aggressive and Defensive
**B**	Aggressive
**C**	Aggressive and Defensive
**D**	Aggressive and Defensive
**E**	Aggressive and Defensive
**F**	Defensive
**G**	Defensive
**H**	Defensive

**Table 2:** Various tyre pressures experimented on.

Notation	Tyre Pressure (psi)
**A**	Front right - 16
	Front left - 15
	Rear right - 14
	Rear left - 14
**B**	Front right - 31
	Front left - 31
	Rear right - 25
	Rear left - 25
**C**	Front right - 33
	Front left - 33
	Rear right - 31
	Rear left - 27
**D**	Front right - 33
	Front left - 33
	Rear right - 26
	Rear left - 26
**E**	Front right – N/A
	Front left - N/A
	Rear right – N/A
	Rear left – N/A

## 2 Experiment Setup

### 2.1 Vehicle experiment setup

**Fig. 1:**
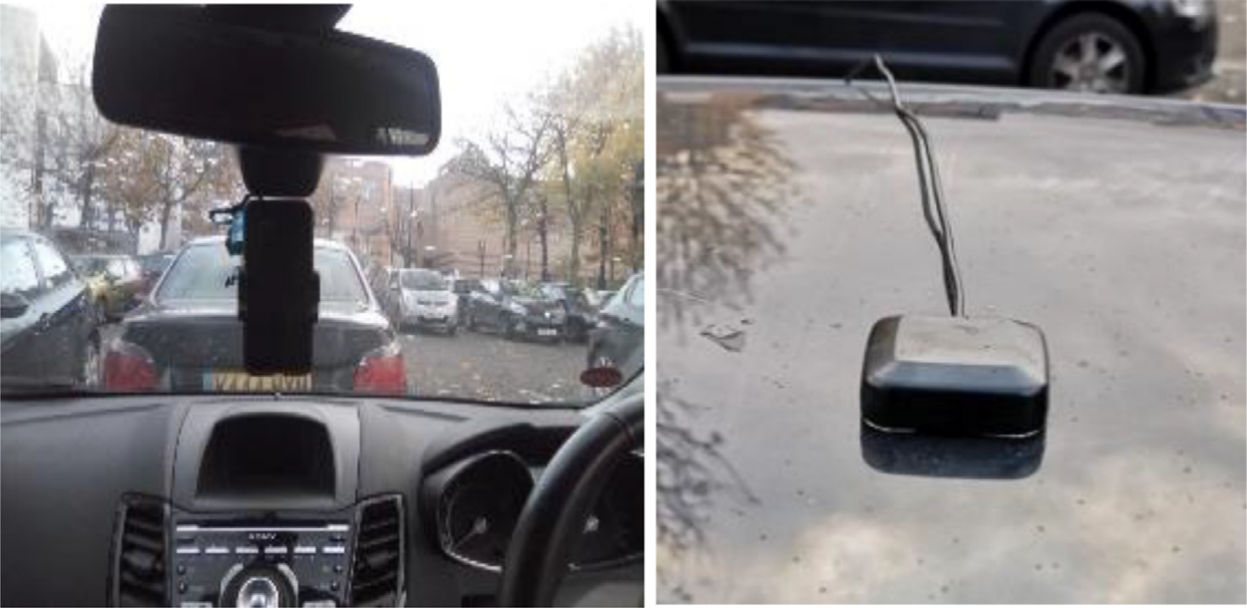
Smartphone and GPS antenna setup.

**Fig. 2:**
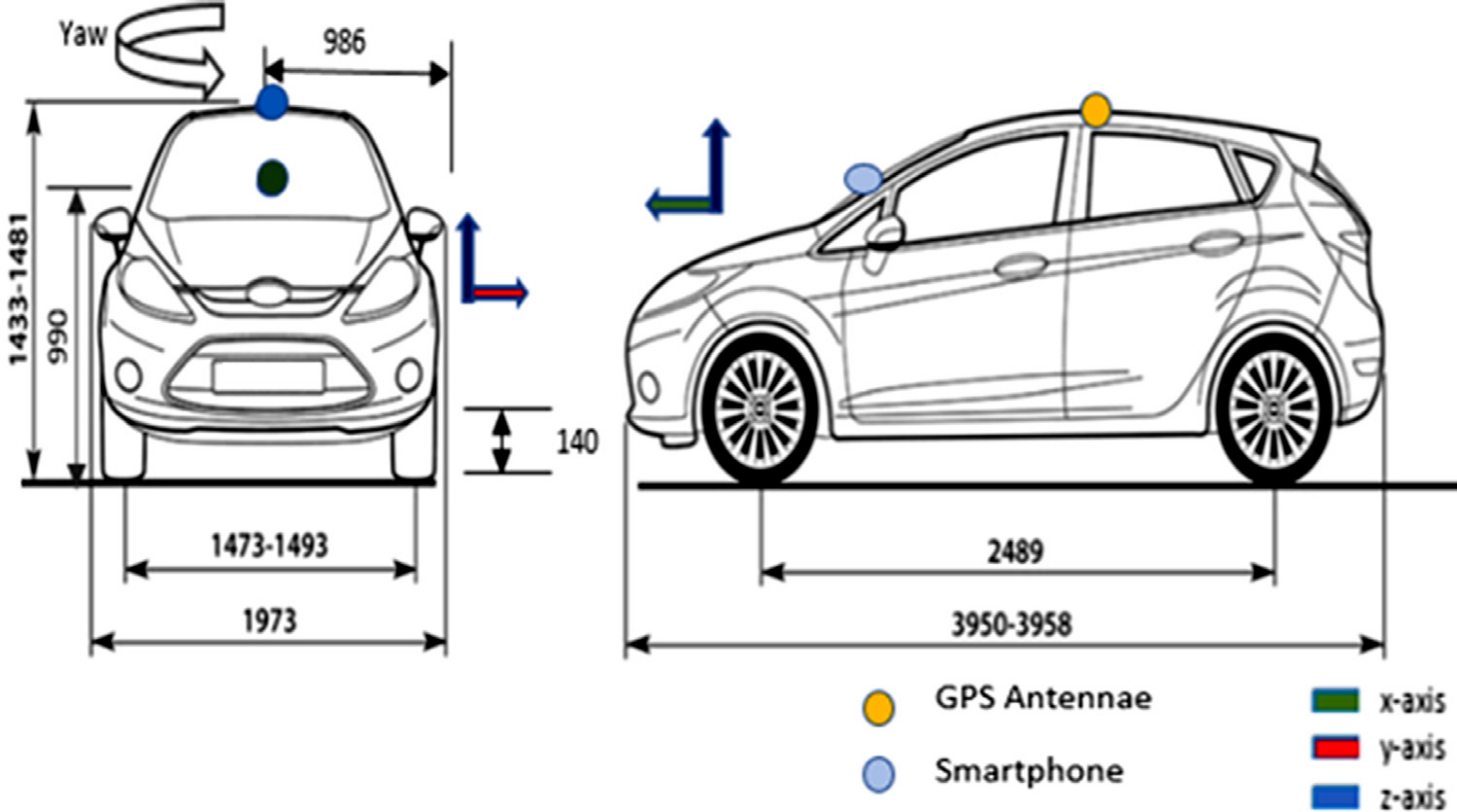
Sensor locations and dimensions of the vehicle [4].

The vehicle used for the data collection exercise was a front wheel drive Ford Fiesta Titanium as shown in Fig. 2. A Racelogic VBOX Video HD2 was used to record the data from the vehicle CAN bus as well as the corresponding GPS coordinates at each sampling instance. As shown in Figs. 1 and 2, the GPS antenna was placed centrally at the top of the vehicle to ensure optimal signal reception. The Racelogic VBOX Video HD2 CAN – Bus data logger (10 Hz) was used to record the data shown in Table 3 directly from the CAN bus of the vehicle with a sampling and update frequency of 10 Hz.

**Table 3:** Information recorded from the Ford Fiesta's ECU.

No	Column Heading	Unit
1	No of GPS satellites available	N/A
2	Time since start of day	seconds
3	GPS Latitude	degrees
4	GPS Longitude	degrees
5	GPS Velocity	km/hr
6	GPS Heading	degrees
7	GPS Height	km
8	GPS Vertical velocity	km/hr
9	Sample period	seconds
10	Steering angle	degrees
11	Wheel speed front left	*rad/sec*
12	Wheel speed front right	*rad/sec*
13	Wheel speed rear left	*rad/sec*
14	Wheel speed rear right	*rad/sec*
15	Yaw rate	*deg/sec*
16	Indicated vehicle speed	km/hr
17	Indicated longitudinal acceleration	g
18	Indicated lateral acceleration	g
19	Handbrake	activated or not (0 or 1)
20	Gear requested	number of gear employed (1–5)
21	Gear	number of gear employed (1–5)
22	Engine speed	rev/min
23	Coolant temperature	degree Celcius
24	Clutch position	activated or not (0 or 1)
25	Brake pressure	psi
26	Brake position	activated or not (0 or 1)
27	Battery voltage	volts
28	Air temperature	degrees Celcius
29	Accelerator pedal position	% activation

**Table 4:** Environmental and driving scenarios investigated.

No	Scenarios
1	Hard-brake
2	Sharp turn left and right
3	Swift maneuvers
4	Roundabout
5	Rain
6	Night and day
7	Skid
8	Mountain/hills
9	Dirt roads/ Gravel roads
10	Country roads
11	Motorway
12	Town-centre driving
13	Traffic congestion
14	Successive left and right turns
15	Varying accelerations within a short duration
16	A -roads
17	B- roads
18	Wet roads
19	U-turns / Reverse drives
20	Mud road
21	Varying tyre pressure
22	Drifts
23	Bumps
24	Inner city driving
25	Winding roads
26	Zig-Zag drives
27	Approximate straight-line motion
28	Parking
29	Potholes
30	Residential roads
31	Stationary (No Motion)
32	Valleys

### 2.2 Smartphone measurement setup

A Ford Fiesta Titanium, Volvo XC70, Renault Mégane and Toyota Corolla Verso were used to collect the smartphone datasets. The smartphone was held with a phone holder attached to the vehicle as shown in Fig. 1. Using the Androsensor app, all data were sampled every 0.1 s with a GPS (smartphone) update rate of 1 Hz. Figs. 1 and 2 show the axis alignment of the smartphone sensors. The smartphone sensors employed were a 3-axis accelerometer, a 3-axis gyroscope, a 3-axis magnetometer and heading, as well as the GPS latitude and longitude coordinates all present within the phone. Other information such as the vehicle's velocity and acceleration were recorded from the smartphone's GPS. Table 5 highlights the data recorded from the smartphone data. The datasets described in Tables A1-1 to A5-2 were collected using the Huawei P20 pro smartphone.

**Table 5:** Information recorded from the smartphone sensors.

No	Column Heading	Unit
1	GPS latitude	degrees
2	GPS longitude	degrees
3	GPS altitude	m
4	GPS speed	km/hr
5	GPS accuracy	m
6	GPS orientation	degrees
7	GPS satellites In range	N/A
8	Time since start	ms
9	Date	YYYY-MO-DD HH-MI-SS_SSS
10	Accelerometer X	m/s²
11	Accelerometer Y	m/s²
12	Accelerometer Z	m/s²
13	Gravity X	m/s²
14	Gravity Y	m/s²
15	Gravity Z	m/s²
16	Gyroscope (Yaw)	rad/s
17	Gyroscope (Pitch)	rad/s
18	Gyroscope (Roll)	rad/s
19	Magnetic field X	µT
20	Magnetic field Y	µT
21	Magnetic field Z	µT
22	Orientation (Yaw)	degrees
23	Orientation (Pitch)	degrees
24	Orientation (Roll)	degrees

**Table A1-1:** Dataset description from Driver A, B and C.

Driver	Dataset name	Features	Cities and towns covered	Weather conditions	Collection date	Velocity and acceleration range	Total time driven and distance covered	Total number of data points	Corresponding smartphone dataset
A	V-S1	B-road (B4101), roundabout (x9), reverse (x5), hilly road, A4053 (ring-road), hard-brake, tyre pressure E	Coventry	15 / 4 °C, Sunny, Humidity:73%, Wind:2.486 mph N	08/09/2019	0.0 to 93.8 km/hr, −0.59 to 0.34 g	86.3 mins, 38.16 km	51,790	S-S1
	V-S2	B-road (B4112, B4065), roundabout (x18), reverse drive (x8), motorway, dirt road, u-turn (x5), country road, successive left-right turns, hard-brake, A-roads (A4600), tyre pressure E	Coventry, Nuneaton	17 / 15 °C Passing clouds. Humidity:47% Wind:3.728 mph N	08/09/2019	0.0 to 105.2 km/hr, −0.56 to 0.43 g	156.5 mins, 75.64 km	93,900	S-S2
	V-S3a	Round-about (x15), u-turn/reverse drive (x4), motorway (M6), A-road (A4600, A426), hard-brake, swift maneuvers, country roads, change in speed, night-time, sharp turn left/right, tyre pressure E	Coventry, Rugby	17 / 12 °C, Passing clouds. Humidity:65% Wind:6.836 mph W	04/09/2019	0.0 to 98.0 km/hr, −0.57 to 0.4 g	41.1 mins, 26.0 km	24,660	S-S3a
	V-S3b	Successive left-right turns (x21), reverse/u-turns (x1), tyre pressure – E	Rugby		04/09/2019	0.0 to 44.8 km/hr, −0.37 to 0.3 g	11.4 mins, 3.8 km	6840	S-S3b
	V-S3c	Roundabout (x4), A-road (A428), country roads, tyre pressure E	Rugby, Coventry		04/09/2019	0.0 to 117.1 km/hr, −0.36 to 0.35 g	62.0 mins, 44.28 km	37,220	S-S3c
	V-S4	Roundabout (x14), u-turn, A-road, successive left-right turns, swift maneuvers, change in speed, night-time, A-road (A429, A45, A46), ring-road (A4053), tyre pressure E	Coventry	13 / 12 °C, Passing clouds. Humidity:83% Wind:8.078 mph WNW	06/09/2019	0.0 to 109.6 km/hr, −0.48 to 0.41 g	163.0 mins, 93.9 km	97,824	S-S4
B	V-M	Roundabout (x30), successive left-right turns, hard-brake (x21), swift maneuvers (x5), country roads, sharp turn left/right, daytime, u-turn (x1), u-turn reverse (x7), tyre pressure E	Coventry	15 / 12 °C, Partly sunny. Humidity:80% Wind:8.078 mph NW	07/09/2019	0.0 to 100.7 km/hr, −1.01 to 0.44 g	176.7 mins, 105.44 km	105,995	S-M
C	V-St1	Roundabout (x9), A-road (A452), B-road, car park navigation, tyre pressure E	Coventry, Kenilworth	13 / 10 °C, Passing clouds. Humidity:56% Wind:7.457 mph ESE	01/04/2019	0.0 to 73.3 km/hr, −0.39 to 0.45 g	95.4 mins, 47.05 km	57,213	N/A

**Table A1-2:** Dataset description from Driver C and D.

Driver	Dataset name	Features	Cities and towns covered	Weather conditions	Collection date	Velocity and acceleration range	Total time driven and distance covered	Total number of data points	Corresponding smartphone dataset
C	V-St4	Roundabout (x1), A-road (A4114, A444, A46), motorway (M40), tyre pressure E	Coventry, Warwick, Chesterton	9 / 4 °C Scattered clouds. Humidity:72% Barometer:991 mbar Wind:12.428 mph W	04/03/2019	0.0 to 101.4 km/hr, −0.27 to 0.13 g	22.7 mins, 28.48 km	13,591	N/A
	V-St6	Motorway (M40), daytime, tyre pressure E	Stokenchurch, Headington Oxford	11 / 9 °C, Passing clouds. Humidity:62% Wind:10.564 mph SSW	05/03/2019	0.0 to 122.1 km/hr, −0.32 to 0.35 g	85.6 mins, 113.63 km	51,360	N/A
	V-St7	Motorway (M40), residential roads, A-road (A46), tyre pressure E	Stokenchurch, Headington Oxford, Coventry, Kenilworth, Warwick	7 / 6 °C Light rain. Partly sunny. Humidity:85% Wind:14.914 mph W	07/03/2019	0.0 to 117.9 km/hr, −0.3 to 0.3 g	74.0 mins, 90.06 km	44,427	N/A
D	V-Y1	Roundabout (x20), successive left-right turns, hard-brake, swift maneuvers, sharp turn left/right, reverse/u-turn (x8), tyre pressure E	Coventry	22 / 16 °C, Passing clouds. Humidity:74% Wind:6.836 mph SSW	30/08/2019	0.0 to 87.5 km/hr, −0.85 to 0.36 g	117.2 mins, 60.86 km	70,341	S-Y1
	V-Y2	Roundabout(x9), u-turn/reverse (x1), A-road, B-road, country road, tyre pressure E	Coventry, Keniltworth	7 / 6 °C Light rain. Partly sunny. Humidity:85% Wind:14.914 mph W	08/03/2019	0.0 to 73.3 km/hr, −0.39 to 0.45 g	95.4 mins, 47.05 km	57,213	N/A

**Table A2-1:** Description of datasets V-Vta1a to V-Vta17 from Driver E.

Driver	Dataset name	Features	Cities and towns covered	Weather conditions	Collection date	Velocity and acceleration range	Total time driven and distance covered	Total number of data points	Corresponding smartphone dataset
E	V-Vta1a	Wet road, gravel road, country road, sloppy roads, roundabout (x3), hard-brake on wet road, tyre pressure A	Nuneaton, Walton on Trent	4–10 / 3–6 °C Passing clouds, Broken Clouds, Scattered Clouds. Humidity:75–93% Wind:4.971 mph SE	14/112,019	0.0 to 103.4 km/hr, −0.54 to 0.35 g	43.0 mins, 40.74 km	25,821	S-Vta1a
	V-Vta1b	Hard-brake on muddy road, wet road, country road, tyre pressure A	Coton in the Elms, Walton on Trent			0.1 to 77.7 km/hr, −0.49 to 0.28 g	1.6 mins, 1.26 km	956	S-Vta1b
	V-Vta2	Roundabout (x2), A-road (A511, A5121, A444), country road, hard-brakes, tyre pressure A	Walton on Trent, Burton on Trent			0.0 to 81.6 km/hr, −0.59 to 0.38 g	18.3 mins, 11.07 km	10,995	S-Vta2
	V-Vta3	Roundabout (x1), swift maneuvers, tyre pressure A	Burton on Trent			0.0 to 45.8 km/hr, −0.31 to 0.27 g	1.5 mins, 0.38 km	875	S-Vta3
	V-Vta4	A-road (A511), tyre pressure A	Burton on Trent			5.9 to 51.7 km/hr, −0.37 to 0.28 g	3.0 mins, 2.02 km	1809	S-Vta4
	V-Vta5	Roundabout (x1), A-road (A511), tyre pressure A	Burton on Trent			29.2 to 51.1 km/hr, −0.26 to 0.09 g	0.6 min, 0.42 km	357	S-Vta5
	V-Vta6	A-road (A511), tyre pressure A	Burton on Trent			43.8 to 103.9 km/hr, −0.24 to 0.13 g	2.3 mins, 2.62 km	1393	S-Vta6
	V-Vta7	Roundabout (x2), A-road (A511), hard-brakes, tyre pressure A	Burton on Trent			22.4 to 113.1 km/hr, −0.54 to 0.18 g	1.4 mins, 1.54 km	857	S-Vta7
	V-Vta8	Town roads, A-roads (A511), tyre pressure A	Hatton Derby			0.0 to 77.6 km/hr, −0.45 to 0.3 g	6.2 mins, 3.43 km	3697	S-Vta8
	V-Vta9	Hard-brakes, A–road (A50), tyre pressure A	Derby			48.9 to 87.7 km/hr, −0.6 to 0.14 g	0.4 min, 0.43 km	226	S-Vta9
	V-Vta10	Roundabout (x1), A-road (A50), tyre pressure A	Sudbury Ashburne			38.8 to 118.0 km/hr, −0.28 to 0.13 g	2.6 mins, 3.95 km	1570	S-Vta10
	V-Vta11	Roundabout (x2), A-road (A50), tyre pressure A	Oaks Green Ashburne			26.8 to 97.7 km/hr, −0.45 to 0.15 g	1.0 min, 0.92 km	589	S-Vta11
	V-Vta12	changes in acceleration in a short period of time, A-road (A515), tyre pressure A	Ashburne			44.7 to 85.3 km/hr, −0.44 to 0.13 g	1.1 mins, 1.27 km	690	S-Vta12
	V-Vta13	A-road (A515), country road, hard-brakes, tyre pressure A	Ashburne			72.7 to 103.6 km/hr, −0.38 to 0.12 g	0.8 mins, 1.14 km	473	S-Vta13
	V-Vta14	Hard-brakes, changes in acceleration in a short period of time, A-road (A515), tyre pressure A	Ashburne			52.8 to 91.0 km/hr, −0.32 to 0.13 g	4.8 mins, 5.45 km	2893	S-Vta14
	V-Vta15	A–road (A515), tyre pressure A	Ashburne			60.1 to 78.8 km/hr, −0.12 to 0.06 g	1.4 mins, 1.72 km	869	S-Vta15
	V-Vta16	Roundabout (x3), hilly roads, country road, A-road (A515), tyre pressure A	Thorpe Ashburne			0.0 to 93.9 km/hr, −0.49 to 0.42 g	18.9 mins, 13.72 km	11,361	S-Vta16
	V-Vta17	Hilly roads, hard-brake, stationary (no motion), tyre pressure A	Ilam, Blore			0.0 to 56.2 km/hr, −0.51 to 0.28 g	7.7 mins, 4.19 km	4594	S-Vta17

**Table A2-2:** Description of datasets V-Vta19 to V-Vta30 from Driver E.

Driver	Dataset name	Features	Cities and towns covered	Weather conditions	Collection date	Velocity and acceleration range	Total time driven and distance covered	Total number of data points	Corresponding smartphone dataset
E	V-Vta19	Hilly road, tyre pressure A	Ilam	4–10 / 3–6 °C Passing clouds, Broken Clouds, Scattered Clouds. Humidity:75–93% SE Wind:4.971 mph	06/112,019	0.0 to 55.2 km/hr, −0.35 to 0.22 g	0.5 min, 0.26 km	310	S-Vta19
	V-Vta20	Hilly road, approximate straight-line travel, tyre pressure A	Ilam			0.0 to 44.8 km/hr, −0.19 to 0.3 g	5.4 mins, 0.39 km	3223	S-Vta20
	V-Vta21	Hilly road, tyre pressure A	Ilam			0.0 to 74.8 km/hr, −0.44 to 0.24 g	3.5 mins, 2.76 km	2088	S-Vta21
	V-Vta22	Hilly road, hard-brake, tyre pressure A	Ilam			14.8 to 55.8 km/hr, −0.53 to 0.16 g	2.6 mins, 1.67 km	1572	S-Vta22
	V-Vta23	Hilly road, hard-brake, tyre pressure A	Thorpe			0.0 to 51.9 km/hr, −0.57 to 0.42 g	1.9 mins, 1.1 km	1119	S-Vta23
	V-Vta24	Hilly road, tyre pressure A	Thorpe			0.0 to 56.4 km/hr, −0.46 to 0.36 g	2.0 mins, 0.71 km	1184	S-Vta24
	V-Vta25	U-turn, tyre pressure A	Thorpe			0.0 to 48.6 km/hr, −0.46 to 0.3 g	1.1 mins, 0.16 km	646	S-Vta25
	V-Vta26	Gravel road, dirt road, hilly road, tyre pressure A	Thorpe			0.0 to 55.1 km/hr, −0.27 to 0.44 g	3.2 mins, 1.02 km	1947	S-Vta26
	V-Vta27	Gravel road, several hilly roads, potholes, country road, A-road (A515), tyre pressure A	Ashburne			0.0 to 65.0 km/hr, −0.43 to 0.29 g	4.8 mins, 3.16 km	2853	S-Vta27
	V-Vta28	Country road, hard-brakes, valley, A-road (A515), tyre pressure A	Milldale			0.0 to 66.0 km/hr, −0.58 to 0.31 g	7.0 mins, 3.94 km	4219	S-Vta28
	V-Vta29	Hard-brakes, country road, hilly road, windy road, dirt road, wet road, reverse drive (x2), bumps, rain, B-road (B5053), country road, u-turn (x3), windy road, valley, tyre pressure A	Wetton, Milldale			0.0 to 102.0 km/hr, −0.8 -to 0.38 g	39.6 mins, 26.12 km	23,737	S-Vta29
	V-Vta30	Rain, wet road, u-turn (x2), A-road (A53, A515), inner town driving, B-road (B5053), tyre pressure A	Buxton			0.0 to 100.0 km/hr, −0.47 to 0.36 g	28.6 mins, 11.77 km	17,179	S-Vta30

**Table A3:** Description of datasets V-Vtb1 to V-Vtb13 from Driver E.

Driver	Dataset name	Features	Cities and towns covered	Weather conditions	Collection date	Velocity and acceleration range	Total time driven and distance covered	Total number of data points	Corresponding smartphone dataset
E	V-Vtb1	Valley, rain, wet road, country road, u-turn (x2), hard-brake, swift manoeuvre, A–road (A6, A6020, A623, A515), B-road (B6405), round about (x3), daytime, tyre pressure A	Bakewell, Tideswell, Ashford on water, Buxton	4–8 / 4 °C Rain, Passing clouds, Broken Clouds, Chilly. Humidity:94–98% Barometer:1004 mbar N Wind:10.564 mph	06/11/2019	0.0 to 101.2 km/hr, −0.63 to 0.36 g	54.1 mins, 41.94 km	32,459	S-Vtb1
	V-Vtb2	Country road, wet road, dirt road, tyre pressure A	Youlgreave			0.0 to 61.1 km/hr, −0.36 to 0.39 g	9.5 mins, 4.35 km	5712	S-Vtb2
	V-Vtb3	Reverse, wet road, dirt road, gravel road, night-time, tyre pressure A	Youlgreave			0.0 to 37.5 km/hr, −0.23 to 0.33 g	13.8 mins, 0.71 km	8289	S-Vtb3
	V-Vtb4	Dirt road, country road, gravel, wet road, tyre pressure A	Youlgreave			0.0 to 32.7 km/hr, −0.31 to 0.27 g	1.0 min, 0.27 km	625	S-Vtb4
	V-Vtb5	Dirt road, country road, gravel road, hard-brakes, Wet road, B-road (B6405, B6012, B5056), inner-town driving, A-road, motorway (M42, M1), rush hour(traffic), round-about (x6), a-road (A5, A42, A38, A615, A6), tyre pressure A	Atherstone, Nuthall, Hilcote, Matlock, Rowsley, Youlgreave			0.0 to 112.9 km/hr, −0.55 to 0.42 g	107.7 mins, 111.66 km	64,610	S-Vtb5
	V-Vtb6	A-road (A5), tyre pressure A	Atherstone			52.7 to 73.0 km/hr, −0.11 to 0.11 g	0.8 min, 0.89 km	508	S-Vtb6
	V-Vtb7	Approximate straight-line motion, night-time, A-road (A5), tyre pressure A	Atherstone			29.1 to 69.2 km/hr, −0.37 to 0.13 g	0.8 min, 0.72 km	461	S-Vtb7
	V-Vtb8	Approximate straight-line motion, nighttime, wet road, A-road (A5), tyre pressure A	Atherstone			60.9 to 76.5 km/hr, −0.35 to 0.08 g	1.2 mins, 1.35 km	699	S-Vtb8
	V-Vtb9	Approximate straight-line motion, night-time, wet road, hard-brakes, A-road (A5), tyre pressure A	Nuneaton			66.8 to 92.0 km/hr, −0.14 to 0.1 g	0.8 min, 0.98 km	457	S-Vtb9
	V-Vtb10	Round-about, wet road, night-time, A-road (A5), tyre pressure A	Nuneaton			26.1 to 58.5 km/hr, −0.24 to 0.12 g	0.3 min, 0.23 km	195	S-Vtb10
	V-Vtb11	Approximate straight-line motion, night-time, wet road, A-road (A5), tyre pressure A	Nuneaton			65.1 to 75.3 km/hr, −0.05 to 0.12 g	0.7 min, 0.84 km	433	S-Vtb11
	V-Vtb12	Roundabout (x1), wet road, night-time, tyre pressure A	Nuneaton			22.2 to 71.6 km/hr, −0.38 to 0.17 g	0.8 min, 0.61 km	490	S-Vtb12
	V-Vtb13	Parking, wet road, tyre pressure A	Nuneaton			7.5 to 43.3 km/hr, −0.31 to 0.22 g	2.1 mins, 0.99 km	1245	N/A

**Table A4-1:** Description of datasets V-Vw1 to V-Vw12 from Driver E.

Driver	Dataset name	Features	Cities and towns covered	Weather conditions	Collection date	Velocity and acceleration range	Total time driven and distance covered	Total number of data points	Corresponding smartphone dataset
E	V-Vw1	Stationary (no motion, sensor bias estimation), daytime, tyre pressure C	Nuneaton	10 °C Smoke. Wind: 6 mph N Humidity: 86%	08/01/2020	0.00 to 0.00 km/hr, 0.00 to −0.00 g	34.1 mins, 0.00 km	20,475	S-Vw1
	V-Vw2	A-road (A5, A421), motorway (M5), daytime, roundabout (x22), u-turn (x2), inner city driving, tyre pressure C	Nuneaton, Hinckley Milton Keynes			0.0 to 115.4 km/hr, −0.62 to 0.45 g	87.9 mins, 98.63 km	52,712	S-Vw2
	V-Vw3	Roundabout (x6), daytime, B-road, inner-city driving, tyre pressure C	Milton Keynes			0.0 to 77.4 km/hr, −0.47 to 0.41 g	6.6 mins, 5.05 km	3942	S-Vw3
	V-Vw4	Roundabout (x77), swift-maneuvers, hard-brake, inner city driving, reverse, A-road, motorway (M5, M40, M42), country road, successive left-right turns, daytime, u-turn (x3), tyre pressure D	Milton Keynes, Buckingham, Droitwich Spa, Kidderminster, Worcester			0.0 to 131.9 km/hr, −0.66 to 0.45 g	211.0 mins, 214.62 km	126,573	S-Vw4
	V-Vw5	Successive left-right turns, daytime, sharp turn left/right, tyre pressure D	Worcester	10 °C Passing clouds. Wind: 2 mph N Humidity: 88%		0.0 to 38.7 km/hr, −0.4 to 0.21 g	1.8 mins, 0.7 km	1050	S-Vw5
	V-Vw6	Bumps, swift-maneuvers, daytime, sharp turn left/right, pressure D	Worcester			3.3 to 40.7 km/hr, −0.34 to 0.26 g	2.1 mins, 1.08 km	1288	S-Vw6
	V-Vw7	Successive left-right turns, daytime, sharp turn left/right, tyre pressure D	Worcester			0.4 to 42.2 km/hr, −0.37 to 0.37 g	2.8 mins, 1.23 km	1689	S-Vw7
	V-Vw8	Successive left-right turns, daytime, sharp turn left/right, tyre pressure D	Worcester			0.0 to 46.4 km/hr, −0.37 to 0.27 g	2.7 mins, 1.12 km	1599	S-Vw8
	V-Vw9	Swift-maneuvers, daytime, hard-brake, tyre pressure D	Worcester			3.8 to 42.0 km/hr, −0.67 to 0.21 g	1.0 min, 0.45 km	601	S-Vw9
	V-Vw10	Hilly road, daytime, pressure D	Worcester			11.8 to 58.9 km/hr, −0.42 to 0.11 g	1.1 mins, 0.74 km	670	S-Vw10
	V-Vw11	Motorway (M5), daytime, roundabout (x5), tyre pressure D				0.0 to 98.4 km/hr, −0.37 to 0.33 g	8.2 mins, 5.85 km	4924	S-Vw11
	V-Vw12	Approximate straight-line motion, daytime, Motorway (M5), tyre pressure D		7 °C Drizzle. Fog. Wind: 5 mph N Humidity: 93%		82.6 to 97.4 km/hr, −0.06 to 0.07 g	1.75 mins, 2.64 km	1050	S-Vw12

**Table A4-2:** Description of datasets V-Vw13 to V -Vw17 from Driver E.

Driver	Dataset name	Features	Cities and towns covered	Weather conditions	Collection date	Velocity and acceleration range	Total time driven and distance covered	Total number of data points	Corresponding smartphone dataset
E	V-Vw13	Approximate straight-line motion, daytime, motorway (M5), tyre pressure D		7 °C Drizzle. Fog. Wind: 5 mph N Humidity: 93%	08/01/2020	94.0 to 115.0 km/hr, −0.07 to 0.06 g	0.5 min, 0.82 km	297	S-Vw13
	V -Vw14a	Motorway (M5), nighttime, tyre pressure D				61.9 to 109.4 km/hr, −0.38 to 0.12 g	5.2 mins, 7.92 km	3140	S-Vw14a
	V -Vw14b	Motorway (M42), nighttime, tyre pressure D				12.6 to 120.1 km/hr, −0.28 to 0.28 g	32.7 mins, 41.21 km	19,600	S-Vw14b
	V -Vw14c	Motorway (M42), roundabout (x2), A-road (A446), nighttime, hard-brakes, tyre pressure D				0.0 to 100.5 km/hr, −0.53 to 0.41 g	26.4 mins, 17.15 km	15,857	S-Vw14c
	V -Vw15	Stationary (no motion, sensor bias estimation), nighttime, tyre pressure D	Dordon	8 °C Cool. Wind: 2 mph N Humidity: 80%		0.0 to 0.0 km/hr, 0.00 to 0.0 g	2.3 mins, 0.00 km	1391	S-Vw15
	V -Vw16a	A–road (A5), roundabout (x2), tyre pressure D	Atherstone	8 °C Rain showers. Overcast. 2 mph N 80%		0.0 to 83.5 km/hr, −0.39 to 0.4 g	10.0 mins, 8.49 km	6000	S-Vw16a
	V -Vw16b	Hard-brakes, nighttime, A-road (A5), approximate straight-line travel, tyre pressure D	Nuneaton			1.3 to 86.3 km/hr, −0.75 to 0.29 g	2.0 mins, 1.99 km	1171	S-Vw16b
	V -Vw17	Hard-brakes, nighttime, A-road (A5), approximate straight-line travel, tyre pressure D	Calcedote			31.5 to 72.7 km/hr, −0.8 to 0.19 g	0.5 min, 0.54 km	329	S-Vw17

**Table A5-1:** Description of datasets V-Vfa01to V-Vfb02c from Driver E.

Driver	Dataset name	Features	Cities and towns covered	Weather conditions	Collection date	Velocity and acceleration range	Total time driven and distance covered	Total number of data points	Corresponding smartphone dataset
E	V-Vfa01	A-road (A444), roundabout (x1), B–road (B4116), daytime, hard-brakes, tyre pressure A	Nuneaton, Twycross, Measham	6 °C Quite cool. Wind: 8 mph N Humidity: 97% 7 °C, Scattered clouds. Wind: 8 mph N Humidity: 87% 5 °C, Light rain. Passing clouds. Wind: 10 mph N Humidity:87%	08/11/2019	0.0 to 98.4 km/hr, −0.56 to 0.42 g	19.2 mins, 18.8 km	11,535	S-Vfa01
	V-Vfa02	B-road (B4116), roundabout (x5), A-road (A42, A641), motorway (M1, M62), high rise buildings, hard-brake, tyre pressure C	Bradford, Measham			0.0 to 117.9 km/hr, −0.67 to 0.48 g	112.9 mins, 163.38 km	67,755	S-Vfa02
	V-Vfb01a	City-centre driving, roundabout (x1), wet road, ring-road, nighttime, tyre pressure C	Bradford			0.0 to 68.9 km/hr, −0.43 to 0.42 g	28.3 mins, 6.81 km	17,000	N/A
	V-Vfb01b	Motorway (M606), round-about (x1), city roads, traffic, wet road, changes in acceleration in short periods of time, nighttime, tyre pressure C				0.0 to 83.0 km/hr, −0.38 to 0.23 g	6.5 mins, 4.07 km	3880	N/A
	V-Vfb01c	Motorway (M62), wet-road, heavy traffic, nighttime, tyre pressure C				0.2 to 104.5 km/hr, −0.36 to 0.38 g	10.5 mins, 10.66 km	6320	N/A
	V-Vfb01d	Roundabout (x1), A-road (A650), nighttime, tyre pressure C				0.0 to 56.0 km/hr, −0.46 to 0.36 g	17.9 mins, 3.39 km	10,713	N/A
	V-Vfb02a	Motorway (M1), roundabout (x2), A-road (A650), nighttime, hard-brakes, tyre pressure D	East Ardsley,	7 °C, Rain showers. Overcast. Wind: 12 mph N Humidity:86%		0.0 to 122.3 km/hr, −0.5 to 0.37 g	59.9 mins, 96.5 km	35,960	N/A
	V-Vfb02b	Roundabout (x1), bumps, successive left-right turns, hard-brakes (x7), swift-maneuvers, nighttime, tyre pressure D	Nuthall			0.0 to 84.3 km/hr, −0.5 to 0.35 g	18.3 mins, 7.69 km	11,000	N/A
	V-Vfb02c	U-turn (x1), hard-brakes, nighttime, tyre pressure D	Nuthall			2.0 to 52.8 km/hr, −0.53 to 0.26 g	1.1 mins, 0.54 km	640	N/A

**Table A5-2:** Description of datasets V-Vfb02d to V-Vfb02g from Driver E.

Driver	Dataset name	Features	Cities and towns covered	Weather conditions	Collection date	Velocity and acceleration range	Total time driven and distance covered	Total number of data points	Corresponding smartphone dataset
E	V-Vfb02d	Round-about (x1), nighttime, tyre pressure D	Nuthall	7 °C, Rain showers. Overcast. Wind: 12 mph N Humidity:86%	08/11/2019	0.0 to 57.3 km/hr, −0.33 to 0.31 g	1.5 mins, 0.84 km	880	N/A
	V-Vfb02e	Changes in acceleration in short period of time, nighttime, tyre pressure D	Nuthall			37.4 to 73.9 km/hr, −0.24 to 0.19 g	1.6 mins, 1.52 km	980	N/A
	V-Vfb02f	Roundabout (x1), nighttime, tyre pressure D	Nuthall			1.6 to 49.5 km/hr, −0.24 to 0.32 g	1.1 mins, 0.47 km	660	N/A
	V-Vfb02g	Motorway (M1), A-road (A42, A444, A5), country road, roundabout (x2), hard-brakes, nighttime, tyre pressure D	Nuneaton			0.0 to 119.4 km/hr, −0.51 to 0.35 g	45.3 mins, 63.56 km	27,159	N/A

## Ethics Statement

The study and data collection have been approved by Coventry University Ethics Board under Project ID P95615.

## CRediT Author Statement

**Uche Onyekpe:** Conceptualization, Methodology, Investigation, Validation, Writing - Original Draft, Writing - Review & Editing, Supervision; **Vasile Palade:** Investigation, Writing - Review & Editing; **Stratis Kanarachos:** Conceptualization, Investigation, Resources, Writing - Review & Editing; **Alicja Szkolnik:** Data Curation, Writing - Review & Editing.

## Declaration of Competing Interest

The authors declare that they have no known competing financial interests or personal relationships which have, or could be perceived to have, influenced the work reported in this article.

## Figures and Tables

**Table A6:** Information on other Smartphone Dataset captured independently from drivers F, G and H.

Driver	Dataset name	Location	Comments	Vehicle model	Phone Model	Total Time driven (mins)	Total distance covered (km)	Total number of data points
F	S-T1, S-T2, S-T3, S-T4, S-T5, S-T6, S-T8, S-T9	France	Information on 3-axis orientation and magnetic field not available.	Renault Megane	Motorola moto G7 power	1005.70	1508.39	603,425
	S-T10, S-T11	France	–	Renault Megane	Motorola moto G7 power	20.60	8.86	12,389
G	S-I	Nigeria	–	Toyota Corolla Verso	Huawei P20 pro,	9.70	0.06	5800
H	S-A1, S-A2, S-A3, S-A4, S-A5, S-A6, S-A7, S-A8, S-A9, S-A10, S-A11, S-A12, S-A13	England	–	Volvo XC70	Blackberry Priv	638.30	1511.93	382,956

## Appendix

Table A1-1–Table A6.

## Footnotes

1 It is difficult to truly determine the centre of gravity of the car under different dynamic conditions, hence the smartphone recording approximates the true motion of the car.

2 Not all “V-” and “S-” dataset were collected simultaneously. All the “V-” datasets without a corresponding “S-” dataset and vice-versa are not placed in the “Synchronised V and S datasets” folder.
